# 术前规划联合荧光胸腔镜精准肺段手术较传统胸腔镜肺段手术治疗早期肺腺癌近期结果比较

**DOI:** 10.3779/j.issn.1009-3419.2021.102.17

**Published:** 2021-07-20

**Authors:** 明然 解, 高祥 王, 美青 徐, 田 李, 世斌 徐, 燃 熊, 巧莉 方

**Affiliations:** 1 230001 合肥，中国科学技术大学附属第一医院胸外科 Department of Thoracic Surgery, The First Affiliated Hospital of University of Science and Technology of China, Hefei 230001, China; 2 230001 合肥，中国科学技术大学附属第一医院手术室 Department of Operating Room, The First Affiliated Hospital of University of Science and Technology of China, Hefei 230001, China

**Keywords:** 肺肿瘤, 肺段切除术, 荧光法, 肺功能, Lung neoplasms, Segmentectomy, Fluorescence, Pulmonary function

## Abstract

**背景与目的:**

肺癌的死亡率居所有恶性肿瘤的第一位，但对于早期肺腺癌患者不同的肺段切除术之间手术效果及对肺功能的影响研究较少。本研究旨在评估术前规划联合荧光胸腔镜精准肺段切除术与传统肺段切除术两种手术方式对早期肺腺癌患者肺功能保留程度和近期结果比较。

**方法:**

前瞻性选取2020年1月1日-2020年10月31日于中国科学技术大学附属第一医院胸外科行胸腔镜肺段切除术患者60例，精准组30例，传统组30例，比较两组患者临床病理特征、围手术期资料和术后肺功能情况。

**结果:**

精准组在手术时间上较传统组更短，差异有统计学意义（*P* < 0.05）。术前肺功能精准组与传统组的用力肺活量（forced vital capacity, FVC）、一秒用力呼气容积（forced expiratory volume in one second, FEV_1_）和一氧化碳弥散量（carbon monoxide diffusing capacity, DLCO）分别为：（3.65±0.63）L *vs*（3.54±0.64）L、（2.72±0.50）L *vs*（2.54±0.48）L及（20.36±3.02）mL/mmHg/min *vs*（19.16±3.18）mL/mmHg/min，差异均无统计学意义（*P* > 0.05）。术后1个月肺功能精准组与传统组的FVC、FEV_1_和DLCO分别为：（3.35±0.63）L *vs*（2.89±0.57）L、（2.39±0.54）L *vs*（2.09±0.48）L及（17.43±3.10）mL/mmHg/min *vs*（15.78±2.86）mL/mmHg/min，差异均有统计学意义（*P* < 0.05）；术后3个月肺功能精准组与传统组的FVC和DLCO分别为：（3.47±0.63）L *vs*（3.20±0.56）L、（19.38±3.02）mL/mmHg/min *vs*（17.79±3.21）mL/mmHg/min，差异均无统计学意义（*P* > 0.05）。

**结论:**

术前规划联合荧光胸腔镜精准肺段切除术在段间平面识别、解剖血管及术后恢复等方便提供了优势，明显缩短了手术时间，使治疗更为精准。

## Abstract (en)

**Background and objective:**

The mortality of lung cancer ranks first among all malignant tumors, but there are few studies on the effect of different segmentectomy on lung function in patients with early lung adenocarcinoma. The purpose of this study was to evaluate the degree of lung function preservation and short-term results of preoperative planning combined with fluorescence thoracoscopic precision segmentectomy and traditional segmentectomy in patients with early lung adenocarcinoma.

**Methods:**

From January 1, 2020 to October 31, 2020, 60 patients underwent thoracoscopic segmentectomy in the Department of Thoracic Surgery of the First Affiliated Hospital of University of Science and Technology of China: 30 patients in precision segmentectomy group and 30 patients in traditional segmentectomy group. The clinicopathological features, perioperative data and postoperative pulmonary function of the two groups were compared.

**Results:**

The operation time of the precision group was shorter than that of the traditional group, and the difference was statistically significant (*P* < 0.05). The preoperative pulmonary function accuracy group and the traditional group in forced vital capacity (FVC), forced expiratory volume in one second (FEV_1_) and carbon monoxide diffusing capacity (DLCO) were (3.65±0.63) L *vs* (3.54±0.64) L, (2.72±0.50) L *vs* (2.54±0.48) L and (20.36±3.02) mL/mmHg/min *vs* (19.16±3.18) mL/mmHg/min, respectively. One month after operation, the FVC, FEV_1_ and DLCO of pulmonary function accuracy group and traditional group were (3.35±0.63) L *vs* (2.89±0.57) L, (2.39±0.54) L *vs* (2.09±0.48) L and (17.43±3.10) mL/mmHg/min *vs* (15.78±2.865) mL/mmHg/min, respectively. Three months after operation, the FVC and DLCO of pulmonary function accuracy group and traditional group were (3.47±0.63) L *vs* (3.20±0.56) L and (19.38±3.02) mL/mmHg/min *vs* (17.79±3.21) mL/mmHg/min, respectively.

**Conclusion:**

Preoperative planning combined with fluorescence thoracoscopic precise segmentectomy provides advantages in intersegmental plane recognition, vascular anatomy and postoperative recovery, which significantly shortens the operation time and makes the treatment more accurate.

近年来，肺癌的发病率在世界范围内呈明显上升趋势，其死亡率始终居于所有恶性肿瘤的第一位^[1-3]^。对于可切除肺癌，传统的治疗模式为以手术为主的综合治疗，肺叶切除+纵隔淋巴结清扫是主流术式。随着低剂量螺旋计算机断层扫描（computed tomography, CT）在肺癌筛查和健康体检人群中的广泛应用，越来越多最大径≤2 cm的早期周围型肺癌获得临床诊断，外科手术方式已不再是一成不变的肺叶切除+纵隔淋巴结清扫术，以肺段切除术为代表的亚肺叶切除获得了广泛的临床关注和应用。越来越多的临床研究的结果显示，对于部分早期肺癌患者，亚肺叶切除术尤其是解剖性肺段切除术可以获得类似于肺叶切除的治疗效果^[4-6]^，并最大程度地保留了患者肺功能。

由于肺段层面血管和支气管变异较大，依据传统解剖学经验的肺段切除容易导致段门小结构误断，其结果导致切除不精准。我们应用虚拟现实规划技术术前明确所有目标靶段的小结构，术中联合应用荧光染色法精确定位段间平面，使手术做到完全精准。从理论上讲，基于虚拟现实术前规划联合术中荧光胸腔镜精准肺段切除相对于传统的肺段切除术具有明显优势，其主要包括围手术期结果、术后并发症、肺功能的保留程度、局部复发和远期预后等指标。但由于该技术应用于国内外临床均不久，临床报道不多。本研究通过前瞻性随机对照临床试验，收集60例早期非小细胞肺腺癌病例，分别给予基于虚拟现实术前规划联合术中荧光胸腔镜精准肺段切除术治疗和传统胸腔镜肺段切除术治疗。比较虚拟现实术前规划联合荧光胸腔镜精准肺段切除术与传统肺段切除术两组患者近期结果和肺功能保留程度。

## 1 资料与方法

### 1.1 临床资料与分组

前瞻性选取2020年1月1日-2020年10月31日中国科学技术大学附属第一医院胸外科行胸腔镜肺段切除术患者60例，其中男35例，女25例，平均年龄为（54.93±9.782）岁。纳入标准：（1）薄层胸部CT见肺部小结节≤2 cm；（2）具备以下其中一个特征：①术后病理证实为原位腺癌；②CT显示磨玻璃成分≥50%；③病灶倍增时间≥400 d；（3）术前无肺功能损害。排除标准：（1）对碘或特定的造影剂过敏者；（2）有心、脑、肺、肾等严重疾病不能耐受检查者；（3）术后病理证实为良性病变。本研究经中国科学技术大学附属第一医院伦理审查委员会批准，患者均签署知情同意书。

按照随机化原则进行分组，进入两个不同的治疗组，以手术方式（基于术前规划联合荧光胸腔镜精准肺段切除治疗或传统胸腔镜肺段切除手术）作为干预，围手术期指标、术后肺功能情况，对入组病例选择不同手术方式，即精准组和传统组。

患者的术前检查包括：血常规、生化、凝血象、免疫组合，心电图、超声心动图，肺功能，气管镜检查，同时行胸部平扫+增强CT明确病变性质，行腹部+双侧肾上腺彩超、头颅磁共振成像（magnetic resonance imaging, MRI）平扫+增强、骨扫描检查以排除远处转移，部分患者行正电子发射计算机断层显像（positron emission tomography-CT, PET-CT）。所有患者的肿瘤分期采用第8版肿瘤原发灶-淋巴结-转移（tumor-node-metastasis, TNM）分期系统。

利用Mimics软件重建的三维模型可以进行动态旋转观察，任意切割显示内部解剖结构，也可以对其进行编辑、修改，便于临床医生更深入细致地对三维重建后的血管分支进行精准定位、定性、定量分析，有助于术前个性化规划和术中精确决策。将胸部薄层（1 mm）增强CT导入该软件，可直观地显示肺部肿瘤的部位、范围及肺段支气管、血管解剖情况，可用于术前制定手术计划和术中导航。除了特殊部位（上叶尖段、舌段、下叶背段等）的结节以外，其余结节术前行CT引导下一次性使用肺结节定位针或硬化剂定位，便于术中精确切除。

### 1.2 手术方法

精准组：患者静脉吸入复合全麻，健侧卧位，单肺通气。采用单孔法（第4或5肋间腋前线与腋中线之间3 cm切口）完成手术。根据术前规划情况解剖出靶肺段动静脉和支气管后离断，使用荧光胸腔镜（欧谱曼迪荧光系统）改为荧光模式，经外周静脉注射吲哚箐绿（丹东医创药业，H20055881）25 mg溶于25 mL灭菌注射用水中，取3 mL经外周静脉快速注入，10 s-15 s后吲哚箐绿经过肺动脉到达肺组织，需切除的靶肺段不显色，其余肺组织则显示为绿色，以电凝钩标记段间面后退出荧光模式，使用切割缝合器进行适形裁剪段间平面。切除肺段过程中，对10、11、12组淋巴结进行采样活检，切除标本后对肺门及纵隔淋巴结进行采样活检，下叶活检第7-9组，左侧上叶活检第5、6组（第4组部分患者活检），右上叶活检第2-4组。送快速冰冻病理，若病灶为浸润性癌或淋巴结为阳性则改行肺叶切除。术后经操作孔放置26号胸腔引流管1根。

传统组：患者静脉吸入复合全麻，健侧卧位，单肺通气。采用单孔法（第4或5肋间腋前线与腋中线之间3 cm切口）完成手术。依据传统的解剖学知识进行肺段门小结构的辨别，离断靶肺段门小结构（动静脉及支气管）后，采用膨胀萎陷法判断段间平面。切除肺段过程中，对10、11、12组淋巴结进行采样活检，切除标本后对肺门及纵隔淋巴结进行采样活检，下叶活检第7-9组，左侧上叶活检第5、6组（第4组部分患者活检），右上叶活检第2-4组。送快速冰冻病理，若病灶为浸润性癌或淋巴结为阳性则改行肺叶切除。术后经操作孔放置26胸腔引流管1根。

### 1.3 观察指标

记录两组患者基本信息，包括年龄、性别、吸烟史、肿瘤位置、肿瘤大小、手术时间、术中出血量、术后并发症、中转开胸例数、胸腔引流管放置时间及住院时间等情况，观察两组患者术前、术后1个月及术后3个月肺功能情况。肺功能包括用力肺活量（forced vital capacity, FVC）、一秒用力呼气容积（forced expiratory volume in one second, FEV_1_）和一氧化碳弥散量（carbon monoxide diffusing capacity, DLCO）。

### 1.4 统计学分析

采用SPSS 24.0统计学软件对数据进行分析。正态分布资料用均数±标准差（Mean±SD）表示，计量资料均数比较采用*t*检验，计数资料比较采用χ^2^检验。*P* < 0.05为差异有统计学意义。

## 2 结果

### 2.1 临床资料比较

本研究共纳入60例患者，均完成胸腔镜肺段切除术，无中转开胸患者，无术后30 d内死亡病例。两组患者年龄、性别、吸烟史、术前肺功能情况、病理类型及肿瘤直径等临床特征之间差异无统计学意义（*P* > 0.05）（表 1）。两组手术肺段解剖切除分布见表 2。

**表 1:** 两组患者临床资料比较 Comparison of clinical data between the two groups

Item	Precision group (*n*=30)	Traditional group (*n*=30)	*t*/χ^2^	*P*
Gender			2.400	0.121
Male	18 (60.0%)	12 (40.0%)		
Female	12 (40.0%)	18 (60.0%)		
Age (Mean±SD, yr)	56.70±10.56	51.80±8.76	1.956	0.055
Smoking history			0.111	0.739
Yes	6 (20.0%)	5 (16.7%)		
No	24 (80.0%)	25 (83.3%)		
Tumor location			0.352	0.950
Right upper lung	12 (40.0%)	10 (33.3%)		
Right lower lung	4 (13.3%)	5 (16.7%)		
Left upper lung	8 (26.7%)	9 (30.0%)		
Left lower lung	6 (20.0%)	6 (20.0%)		
Pathological type			0.314	0.855
AAH	4 (13.3%)	3 (10.0%)		
AIS	16 (53.4%)	18 (60.0%)		
MIA	10 (33.3%)	9 (30.0%)		
Tumor size (Mean±SD, cm)	0.93±0.30	0.88±0.39	0.551	0.584
AAH: atypical adenomatoid hyperplasia; AIS: adenocarcinoma *in situ*; MIA: microinvasive adenocarcinoma.				

**表 2:** 两组患者手术肺段切除分布 Distribution of segmental pneumonectomy in two groups of patients

Item		Precision group (*n*=30)	Traditional group (*n*=30)
Right upper lung	S1	8	4
	S2	3	2
	S3	1	4
Right lower lung	S6	1	3
	S7	0	1
	S8	1	1
	S9	1	0
	S10	1	0
Left upper lung	S1+S2	5	7
	S3	1	1
	S4+S5	2	1
Left lower lung	S6	4	5
	S7+S8	1	1
	S9	0	0
	S10	1	0

### 2.2 围手术期资料比较

精准组在手术时间上较传统组更短，差异有统计学意义（*P* < 0.05）（表 3）。精准组在术中出血量、术后引流量、术后胸管留置时间和术后住院时间方面较传统组差异无统计学意义（*P* > 0.05）（表 3）。两组患者在淋巴结清扫的站数和枚数方面差异无统计学意义（*P* > 0.05）（表 3）。两组患者在24 h和72 h术后数字评分法（numerical rating scale, NRS）评分方面差异无统计学意义（*P* > 0.05）（表 3）。

**表 3:** 两组患者围手术期比较（Mean±SD） Perioperative comparison between the two groups (Mean±SD)

Index	Precision group (*n*=30)	Traditional group (*n*=30)	*t*	*P*
Intraoperative bleeding volume (mL)	23.17±13.80	28.00±17.30	-1.196	0.236
Postoperative hospital stay (d)	4.63±2.12	5.03±1.82	-0.781	0.438
Operation time	113.07±23.78	142.33±52.78	-2.769	0.008
Postoperative drainage (mL)	270.33±174.26	294.83±169.55	-0.552	0.583
Postoperative time with tube (d)	3.70±1.36	4.10±1.78	-0.973	0.335
Number of lymph node dissection	4.83±3.99	4.57±5.217	0.222	0.825
Number of lymph node dissection stations	2.30±1.51	2.20±1.73	0.238	0.812
Postoperative 24 h NRS score	3.53±0.62	3.70±0.65	-1.008	0.317
Postoperative 72 h NRS score	2.87±0.68	2.97±0.89	-0.489	0.627
NRS: numerical rating scale.				

### 2.3 肺功能相关情况

术前，肺功能精准组与传统组FVC、FEV_1_和DLCO的差异均无统计学意义（*P* > 0.05）。术后1个月，肺功能精准组与传统组FVC、FEV_1_和DLCO的差异有统计学意义（*P* < 0.05）；术后3个月，肺功能精准组与传统组FVC和DLCO的差异无统计学意义（*P* > 0.05），但FEV_1_的差异有统计学意义（*P* < 0.05）（表 4）。

**表 4:** 两组患者术前术后肺功能指标比较（Mean±SD） Comparison of pulmonary function indexes between the two groups before and after operation (Mean±SD)

Time	Pulmonary function index	Precision group (*n*=30)	Traditional group (*n*=30)	*t*	*P*
Before operation	FVC (L)	3.65±0.63	3.54±0.64	0.658	0.513
	FEV_1_ (L)	2.72±0.50	2.54±0.48	1.396	0.168
	DLCO (mL/mmHg/min)	20.36±3.02	19.16±3.18	1.506	0.137
1 month after operation	FVC (L)	3.35±0.63	2.89±0.57	2.930	0.005
	FEV_1_ (L)	2.39±0.54	2.09±0.48	2.270	0.027
	DLCO (mL/mmHg/min)	17.43±3.10	15.78±2.86	2.135	0.037
3 months after operation	FVC (L)	3.47±0.63	3.20±0.56	1.716	0.091
	FEV_1_ (L)	2.55±0.49	2.29±0.48	2.016	0.048
	DLCO (mL/mmHg/min)	19.38±3.02	17.79±3.21	1.970	0.054
FVC: forced vital capacity; FEV_1_: forced expiratory volume in one second; DLCO: carbon monoxide diffusing capacity.					

## 3 讨论

随着现代腔镜技术的进步，早期肺癌的治疗方式不再是一成不变的肺叶切除术加纵隔淋巴结清扫术。目前，以肺段切除术为代表的亚肺叶切除获得了临床广泛的关注和应用。另外，越来越多的回顾性研究^[7-9]^报道，对于部分早期非小细胞肺癌，肺段切除术并没有增加局部复发率，同时可以达到相似的5年生存率。目前国内外，除少数大医院根据术前三维重建技术指导术中解剖外，绝大多数医院仅根据传统的解剖学知识施行肺段切除手术。这种非精准肺段切除手术存在以下弊端：①在肺段层面的解剖方面，个体差异较大，每例患者的血管和支气管的段及亚段走向不尽相同。因此，仅根据传统的解剖学知识来进行术中解剖是不够的，往往容易误断或少断；②血管和支气管的误断或少断，术中会导致段间平面的错判，进而导致病灶切除范围不够或过大，无法做到精准切除；③术中肺段动静脉、支气管和段门平面切除不精准，术后会导致局部肺段的不张，甚至咯血等较严重的并发症发生，未能达到保留更多肺功能的目的；④肺复张不良会导致术后复查胸部CT肺部阴影长期不能消退，导致患者怀疑治疗效果；⑤同一肺段不同肺段之间的血管存在一些交通支，这导致传统的膨胀萎陷法判断段间平面等待时间过长，有时甚至不能显示其平面。近年来，三维重建技术及虚拟手术规划已广泛应用于肝胆外科、骨科和整形外科等的临床诊疗^[10, 11]^。目前，国外肺部三维重建技术及虚拟手术规划已用于对胸腔镜解剖性肺段切除术的术前病情评估、模拟肺段切除及制定手术方案、术中导航等的研究^[12, 13]^，国内则应用较少，且主要用于指导胸腔镜解剖性肺段切除术^[14, 15]^。本研究发现：术前规划联合荧光胸腔镜精准肺段切除术较传统肺段切除术在手术时间、术后肺功能保留方面更好。进一步论证了精准治疗在肺段手术中的优势。

本研究发现术前规划联合荧光胸腔镜精准肺段切除术在手术时间方面明显优于传统肺段手术。邵丰等^[16]^通过回顾性分析20例荧光法与40例膨胀萎陷法行胸腔镜解剖性肺段切除术非小细胞肺癌患者临床资料发现，荧光法较膨胀萎陷法段间平面出现的更快，手术时间更短。张彤等^[17]^通过回顾性分析60例荧光法与97例膨胀萎陷法行胸腔镜解剖性肺段切除术非小细胞肺癌患者临床资料发现，荧光染色组的段间平面形成时间更早、手术时间缩短。我们认为其主要原因在于：①荧光法识别段间平面快，从注射吲哚菁绿到出现段间平面一般在10 s左右，而传统使用的膨胀萎陷法识别段间平面一般需要15 min左右，因此精准组在识别段间平面方面节约了手术时间；②膨胀萎陷法需要将靶肺段充气膨胀呈现段间平面，而荧光法无需将患肺膨胀；③精准组术前重建肺部结节的部位、范围及肺段支气管、血管解剖情况，术中极大地减少了手术医师对血管解判断的时间，从而能够更快更精准的完成手术。

从理论上讲，术前规划联合荧光胸腔镜精准肺段切除术除相对于传统的肺段切除术具有明显优势，其中包括术后肺功能保留程度。术后肺功能决定肺部手术患者术后生活质量。本研究发现，精准肺段切除组术后1个月肺功能较传统肺段切除组更好。但是，术后3个月两组术后肺功能几乎无明显差异。但由于该技术应用于国内外临床均不久，尚未见临床研究报道该方面内容。我们认为造成这种结果的原因有以下几种可能：①三维重建及虚拟手术规划应用于术中影像导航辅助胸腔镜解剖性肺段切除手术，可避免损伤或误断肺段支气管和血管，从而使保留肺段的肺功能影响最小化；②术中采用荧光胸腔镜判断段间平面，减少了肺段间交通支的干扰，而传统使用膨胀萎陷法显现段间平面存在误差较大及个体差异大等问题，从而使术后肺功能可能产生一定差异；③随着患者恢复时间的增加及患者肺功能的锻炼，残余肺组织的代偿及膨胀，使患者肺功能进一步恢复，造成传统肺段切除术术后患者肺功能与精准肺段切除术患者无明显差异。

总而言之，术前规划联合荧光胸腔镜精准肺段切除术在段间平面识别及解剖血管等方便提供了优势，给术者提供了良好的体验。从术后肺功能方面来看，术前规划联合荧光胸腔镜精准肺段切除术与传统肺段手术对肺功能保留在术后短时间内可能存在差异，但随着患者术后恢复时间的增加，肺功能并无明显差异。另外，本研究存在样本量小、随访时间短等不足情况，需要进一步大样本，远期随访情况等相关研究，以探讨术前规划联合荧光胸腔镜精准肺段切除术在治疗非小细胞肺癌方面的价值及优势。
